# Dielectric Study of Liquid Crystal Dimers: Probing the Orientational Order and Molecular Interactions in Nematic and Twist-Bend Nematic Phases

**DOI:** 10.1021/acs.jpcb.3c03496

**Published:** 2023-08-01

**Authors:** Antoni Kocot, Małgorzata Czarnecka, Yuki Arakawa, Katarzyna Merkel

**Affiliations:** † Institute of Materials Engineering, Faculty of Science and Technology, University of Silesia, 75 Pułku Piechoty 1a, Chorzów 41-500, Poland; ‡ Faculty of Electrical Engineering, Automatics, Computer Science and Biomedical Engineering, AGH University of Science and Technology, al. Adama Mickiewicza 30, Cracow 30-059, Poland; § Department of Applied Chemistry and Life Science, Graduate School of Engineering, Toyohashi University of Technology, Toyohashi 441-8580, Japan

## Abstract

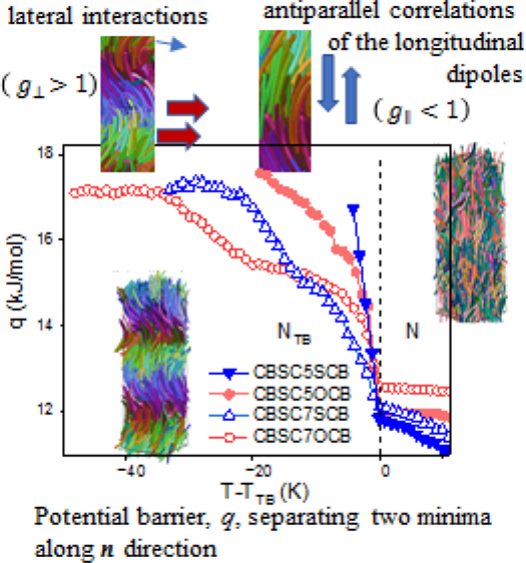

Dielectric spectroscopy in frequencies that range from 10 Hz to 1 GHz has been used to study the molecular orientational dynamics of the two types of dimers that form the twist-bend nematic phase over a wide range of temperatures for both nematic and twist-bend nematic phases. The symmetrical and asymmetrical liquid crystal dimers with the cyanobiphenyl mesogenic groups were investigated. The results were analyzed within the framework of the molecular theory of dielectric permittivity for nematogens. The two molecular processes can be assigned to the reorientation of the monomeric unit: the high frequency one to the precessional rotation of the longitudinal components of the cyanobiphenyl groups (CN) and the second (low frequency) to the end-over-end rotation of the CN dipole around the short molecular axis. The precession mode, which is determined by the local order and is almost unaffected by the phase transition from the nematic to the twist-bend phase, while the end-over-end rotation clearly slowed down at the transition, as it is affected by the growth of the intermolecular interactions. The latter corresponds well to the fact revealed by IR spectroscopy about the longitudinal correlation of the molecular dipoles.

## 1 Introduction

It has been commonly accepted that the large scale structures and dynamics of soft matter are controlled by weak intermolecular forces on the order of thermal energy. Therefore, thermal fluctuations are sufficient to significantly change the structure of a material. For entropic reasons, macromolecules often exhibit a spontaneous self-organization into complex, ordered structures. These structures in soft matter systems can occur hierarchically and can range from length scales that are comparable to the size of a single molecule to the higher order, super-molecular scales. Typical examples are the spontaneously formed nanometer-sized patterns that are found in block-copolymer systems, or the hierarchical assembly of lipid molecules into membranes in biological materials. A crucial factor for determining the character of an ordered structure is its mesogenic shape.^1^ Rod-shaped, disc-shaped, bent-core, or umbrella-shaped molecular forms are just a few examples of mesogenic design. Bent-core mesogens, for example, exhibit a rich variety of polar phases, which are not typical for rod-shaped mesogens. The unusual properties of the bent-core nematic phases of the resorcinol derivatives, such as the formation of optically active domains, which enhanced their flexoelectric effect^2^ and strong magneto-optical effects,^3,4^ have been reported. Conversely, a spontaneous self-assembly can provide information on the assembly of the components, that is, their conformation/shape, charge, polarizability, magnetic dipole, mass, and so forth, in a specific phase, as these properties determine their interactions. Molecular self-assembly studies provide essential information about the influence of intermolecular interactions on the structure of the investigated molecular system. Therefore, such studies are crucial when anisotropic molecular systems can lead to direction-dependent physical properties. Helical structures, which have been the subject of great interest in the field of molecular sciences partly because of their potential applications in electro-optical devices, have emerged as one of the most important subgroups of hierarchical structures.^5−8^ Even when chiral nematic phase has a pitch of few molecular lengths, the director twists and bends are generally accepted, as has been confirmed by several experiments: polarizing optical microscopy (POM),^9^ freeze fracture transmission electron microscopy,^10,11^ atomic force microscopy,^11^ nuclear magnetic resonance (NMR),^12,13^ Raman scattering,^14^ IR spectroscopy (IR),^15,16^ and non-resonant hard X-ray scattering,^17,18^ as well as C K-edge and Se K-edge resonant X-ray scattering.^19,20^ The chirality of the phase is measured directly using the synchrotron circular dichroism of aligned samples.^21^ However, the specific structure of the mesophase still remains a matter of debate.^22−24^ Although the basic features of this phase are now established, the nature of this phase is still discussed controversially. The existing materials do not behave fully in line with the prevailing models.^8,10,17,18^ Besides, there have been significant theoretical and simulation efforts toward developing an understating of the interactions and underlying mechanisms that account for the origin of such nematic–nematic (N–N) transitions, with most recent results indicating steric interactions and the primary role of the molecular shape as the driving force for the phase formation.^15,16,25−27^

The primary motivation for this work was to analyze the orientational dynamics of molecules in the nematic phases: N and twist-bend (N_TB_) using dielectric spectroscopy of the bent-shape liquid crystals. The collective dynamics were studied already in ref (28); here, we discuss only the molecular modes in nematic (N and N_TB_) phases, which are formed by the polar symmetric dimers CBSCnSCB and the similar asymmetric dimers CBSCnOCB. Dielectric relaxation measurements were conducted for both samples in the entire temperature range up to the point of glass transition. The observed relaxation processes were interpreted based on the theoretical models. In both nematic phases, the dipolar–dipolar groups that are associated with the terminal cyanobiphenyl (CB) groups led to two relaxation modes that were related to the rotational dynamics of the molecules. The low-frequency mode, which is denoted as **m_1_**, was caused by end-to-end motion of the dipolar groups that are parallel to the mesogen axis. The high-frequency mode, which is denoted as **m_2_**, was the result of the precessional rotation of the dipolar groups around the director. These modes contributed to the complex dielectric permittivity differently depending on the orientation of the director relative to the electric field that was being measured. The second important part of the analysis was the study of the glass transition, which was clearly identified below the N–N_TB_ transition in both longer dimers. The glass transition is characterized by the temperature *T*_g_ and the rate at which various properties change with temperature as the liquid phase approaches the glass transition. For systems that exhibit a certain number of structural relaxations, it is possible to identify the dielectric glass transitions that correspond to each of the two types of molecular motion. The results for the symmetrical and asymmetric liquid crystal dimers presented here are discussed to provide information on how the changes in the molecular interactions that are observed near the N–N_TB_ transition and below it affect the aggregation process and how they are frozen during glass formation.

## 2 Experimental Section

### 2.1 Materials

**Figure 1:**
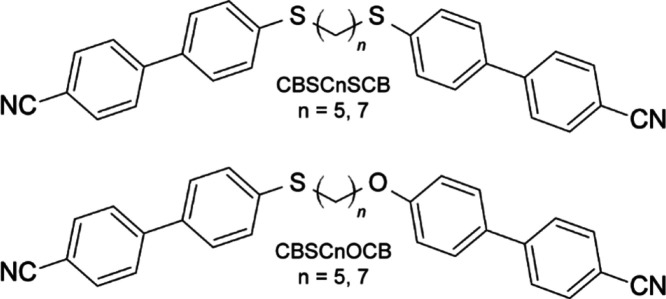
Molecular structure of symmetrical (CBSCnSCB) and asymmetrical (CBSCnOCB) liquid crystal dimers with the cyanobiphenyl mesogenic groups.

The symmetrical and asymmetrical liquid crystal dimers with the CB mesogenic groups were investigated. The symmetric dimers with the acronym CBSCnSCB contain thioether bridge (S–Cn–S) with five/seven methylene groups in the linker (*n* = 5, 7). In the asymmetric dimers with the acronym CBSCnOCB (*n* = 5, 7), the mesogens are linked to an alkyl chain with five or seven methylene groups on one side by a thioether bridge and on the other by an ether bridge. The molecular structure of the investigated compounds is shown in Figure 1. Synthesis details concerning the thioether/ether dimers were described in refs (29, 30).

### 2.2 Dielectric Measurements

Dielectric spectroscopy studies were performed in the range from 10 Hz to 1 GHz using two impedance/network analyzers, HP4I92A and HP4I95A, measured on planar and homeotropic aligned cells. A number of cells with cell gaps varying from 1.6 to 12 mm were tested. These include planar commercial cells with SE-130 polymer aligning (Nissan Chemical Industries, Ltd). For the higher frequency dielectric measurements, gold-plated cells were applied. The temperature of the sample was stabilized using a PID temperature controller within ±2 mK. For the high-frequency range, we use a parallel plate capacitor made of two circular gold-plated brass electrodes with 5 mm diameter separated by silica spacers. The sample was placed at the end of a coaxial line. The planar alignment in the range of the N and N_TB_ phases was controlled by observing the strength of the high frequency mode.

## 3 Theoretical Basis

### 3.1 Dielectric Properties of the Molecular System

Electric dipoles can be used as effective molecular probes to detect changes in the molecular structure using dielectric measurements. The electric permittivity is by definition an II-order tensor quantity, which relates the induced polarization *P* to the macroscopic electric field *E*.
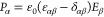
1

Generally, a symmetric II-order tensor has six independent components, which can be reduced to three diagonal components if the tensor is defined in the principal axis system. For axial symmetry, there are only two independent components of the permittivity: parallel and perpendicular to a single axis of symmetry. Under such assumptions, the dielectric permittivity tensor can be expressed by the mean value ε and anisotropy Δε.
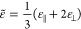
2
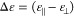
3

Dielectric permittivity is a measure of the averaged molecular mean-square dipole moment, which is determined by the conformationally averaged sum of the dipole component vectors and is therefore directly related to the distribution of molecular conformations.^31−33^ In conclusion, we can state that electric dipoles can be used as effective molecular probes to detect changes in molecular structure by means of dielectric measurements.

For anisotropic materials, the direction of the measurement is defined by the topology of the sample, and where the electrodes are situated. For a fluid, these directions are determined by external considerations such as the surface area, flow ordering, or the electric or magnetic fields. The ordering of a uniaxial liquid crystal is determined by a single director and can be arranged parallel or perpendicular to the surface of the electrodes via the appropriate surface preparation.

To represent the relationship between the dielectric properties of a liquid crystal and the molecular properties of a system of molecules, it is possible to obtain results that are equivalent to Onsager’s equation, but only in the case of an anisotropic fluid. This was first done for a uniaxial system by Maier and Meier.^34^ The following equations give the dependencies of the dipolar contribution on the main order parameter, *S*, and the frequency of the field, ω.
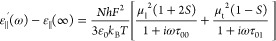
4a
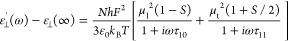
4b

These equations assume that both the cavity and its reaction field are isotropic, thus having a value that is the same as for an isotropic medium. There is some basis for this assumption because the anisotropy of an external field reflects the far-field anisotropy in a radial system; for the radial distribution function for molecules, however, many models consider the development of an anisotropic internal field in liquid crystals, and these can be used if necessary.

Generally, the anisotropy of a material appears through the microscopic order parameters *S*, *P*, *D*, and *C*, which determine the degree of the ordering of molecules due to the phase symmetry. The basic order parameter is the *S* parameter, and it determines the ordering of the molecular axes with respect to the principal director. If the liquid crystal molecules are biaxial, but the phase is uniaxial, then it is necessary to introduce the *D* parameter. For a biaxial molecule, the two *x*, *y* axes that are perpendicular to the long molecular axis behave differently, and therefore, the ordering of these two axes with respect to the uniaxial director of the sample will also be different. The difference between them will be precisely the parameter *D*.

The Maier–Meier equations presented here for the low-frequency components of permittivity represent the dependencies on all of the order parameters. These equations predict that the average value of the permittivity should be independent of the orientation order. Disregarding any density changes, the development of this value should be continuous through all of the liquid crystal phases. These predictions are not always confirmed by experiments for dipole mesogens, but are often observed for the mean value ε̅ with phase changes, including in the transition into isotropic liquids.

Dielectric measurements of liquid crystals provide an opportunity to observe the organization of molecules and changes in the components in permittivity during phase transitions of a liquid crystal between the nematic phases and from the nematic (N) phase to the various smectic (Sm) phases. Thus, they primarily reflect changes in the orientational order and changes in the symmetry.

In liquid-crystalline dimers, the molecular flexibility that is provided by the changing distribution of the conformers has a strong influence on the energetically favored molecular shapes and thus on the mesophase properties.^35,36^ The conformational distribution is also influenced by the anisotropic nematic interactions, thus favoring the molecular geometries that can better adapt to the nematic ordering and environment. The temperature dependence of the dielectric response of a liquid crystalline phase depends on the rotational distribution of the molecular dipoles in the presence of an electric field and the orientation of the phase. However, the order parameter itself is temperature-dependent and, as indicated earlier, the conformational distribution affects via both the shape and the order parameter.

The effect of the macroscopic anisotropy in measuring the dielectric properties of an LC material was calculated for a model of a polarizable cavity that was immersed in a dielectric continuum. To go further beyond this approach, it was necessary to create a model for the anisotropic local field acting on the molecule and for the short-range interactions between the dipole moments inside the cavity. The former depends on the long-range anisotropy in the radial distribution, while the dipole–dipole interaction can be described by the anisotropic Kirkwood coefficient that is defined for the different directions in the sample. These coefficients are most easily defined as the corresponding dipole correlation functions, where *i* is the subscript for parallel and perpendicular directions.
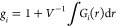
5
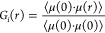
6

Thus, *g*_∥_ (parallel) and *g*_⊥_ (perpendicular) determine to what extent the individual dipole moment projections are correlated along the major axis of anisotropy, assuming the uniaxial case. If the rotational motion of the molecules is isotropic, then the correlation coefficients along different axes should be the same.

### 3.2 Dielectric Relaxation Times

The simplest dielectric absorption model that is applicable to most materials is the Debye model, which describes the single dipole relaxation process. The temperature dependence of the relaxation time can be represented by the phenomenological Arrhenius equation, which introduces the activation energy *E*_a_ for the reorientation of a molecular dipole in a dielectric environment.
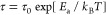
7

Although the Arrhenius equation describes the dielectric relaxation of many simple fluids well, there are several materials for which the equation fails, and it is necessary to modify equations that describe experimental results. One of the phenomenological equations that are most commonly used to describe the temperature dependence of relaxation time data (τ) is the Vogel–Fulcher–Tammann (VFT) equation:
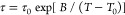
8

Despite some concerns about the validity of the VFT equation, because it assumes a dynamic divergence of the relaxation time at some finite temperature, *T*_0_, some theories such as the Adam–Gibbs entropy model^37^ and some more recent theoretical models^38−40^ are based on the VFT equation. The basic idea behind these theories is that glass formation is associated with highly cooperative movements in a structurally fluctuating sample, with cooperativity growing as *T*_g_ is approached.

### 3.3 Molecular Modes in Liquid Crystals

This problem is compounded in dielectric studies of liquid crystals, because of their macroscopic anisotropy and the presence of a nematic potential. One solution of the rotational diffusion equation for a rigid dipolar molecule in the presence of a nematic potential predicts that the frequency dependence of each component of the electric permittivity can be characterized by two exponential decays.^41,42^ Rotation of the molecule about a short molecular axis in the presence of the nematic potential gives rise to the low frequency relaxation mode **m_1_** that is detected in the parallel component of the permittivity. Relaxation of the longitudinal component of the molecular dipole by precession about the director axis contributes to a high frequency mode **m_2_** that is detected in the perpendicular component of the permittivity. The dielectric relaxations were related to the rotational diffusion of the electric dipoles: the reorientation of the end-to-end longitudinal dipoles at low frequencies (**m_1_**) and the precessional motion of the dipolar groups around the director for the high-frequency branch of a spectrum (**m_2_**).

A nematic potential is assumed to be of the form:
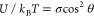
9
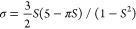
10where σ is the nematic potential barrier parameter, defined as σ = *q*/*RT*, and *q* is the height of the barrier separating the two minima along the *n* direction, while the approximate expressions for the relaxation times were derived as reported in ref (42). The dielectric relaxations were related to the rotational diffusion of the dimer molecules: the reorientation of the end-to-end dipolar groups parallel to the director at low frequencies (**m_1_**) according to the theoretical model of dielectric relaxation in nematic dimers,^33^ and the precessional motion of the dipolar groups around the director for the high-frequency branch of a spectrum (**m_2_**). The longitudinal, μ_l_, and transverse, μ_t_, components of the molecular dipole moment, μ, contribute to the dielectric permittivity differently, and they relax at different frequencies of the probe field. In solving the rotational diffusion equation, a uniaxial nematic potential is assumed. The relaxation times of modes **m_1_** and **m_2_** are described, in terms of spherical harmonics, by τ_00_ and τ_10,_ respectively_:_
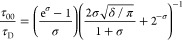
11
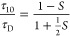
12The relaxation time, τ_D,_ is the one for rotational diffusion in the isotropic phase.

## 4 Results and Discussion

### 4.1 Electric Permittivity of Dimers

Dielectric permittivity is a measure of the averaged molecular mean-square dipole moment, which is determined by the conformationally averaged sum of the dipole component vectors and is therefore directly related to the distribution of the molecular conformations.^31−33^ Therefore, electric dipoles can be used as effective molecular probes to detect changes in the molecular structure using dielectric measurements. For CB7CB and CB9CB symmetric dimers, the mean square dipole moment is determined by the vector-averaged sum of the dipole moments that are associated with the nitrile groups that are attached to the terminal mesogenic units. Different molecular conformers will contribute to the measured components of dielectric permittivity differently.

In liquid-crystalline dimers, the molecular flexibility that is delivered by the linkage and its conformational mobility has a strong influence on the energetically selected molecular appearances and thus on the mesophase properties.^35,36^ To some extent, the conformational distribution is also determined by the nematic interactions, thus approving geometries that can be better adapted to nematic ordering. The temperature dependence of the dielectric response of a liquid crystalline phase depends on the rotational distribution of the molecular dipoles in the presence of an electric field as well as the orientation of the phase. However, the order parameter itself is temperature-dependent and, as was indicated earlier, the conformational distribution affects both of the molecular dipoles via the shape and the order parameter. The results of calculating the conformational energy as a function of the bending angle of a dimer indicated that the CB7CB homolog had two peaks: a strong one that was centered around 120° (extended conformers) and a weaker peak at 30° (hairpin conformers).^31^ They also showed that as the orientational order increased, the population of extended conformers grew; simply, the shape of the molecule adapted to the increased orientational order in the nematic phase while cooling.^43^ As was expected, the hairpin conformers had a large contribution to the parallel permittivity of the mean-square dipole moment that was measured along the director. The extended conformer had a zero mean-square dipole component, thus contributing to parallel permittivity along the dipole. However, for such conformers, there was a non-zero (transverse) contribution of the dipoles to the perpendicular component of permittivity, which was dependent on the angle between the end dipolar groups. Because of the large amount of molecular flexibility, each of the dipolar group had a large contribution to square dipole moment. Another important phenomenon appeared at the transition to the N_TB_ phase. Because of the specific arrangement of the terminal mesogenic units in the local environment, the short-range interactions between dipole moments increased, which affected both the effective dipole moment through the positional correlations, and, consequently the dielectric permittivity. In addition, the relaxation rates for the transverse and longitudinal dipole polarization were different, and therefore, the changes in the conformational distribution of the interarm angle were clearly reflected by the dielectric permittivity measurements. We measured the parallel and perpendicular components of the static dielectric permittivity when cooled from the isotropic phase. The perpendicular and parallel components of the permittivity (ε_⊥_, ε_||_) were obtained in the planar and homeotropic orientations, respectively, in the presence of a weak measuring field (0.5 Vrms/μm). The opportunity of obtaining a homeotropic orientation in a strong field was rejected, as this clearly evokes the conformational changes that are induced by the field. Both components were measured as a function of temperature at different frequencies.

**Figure 2:**
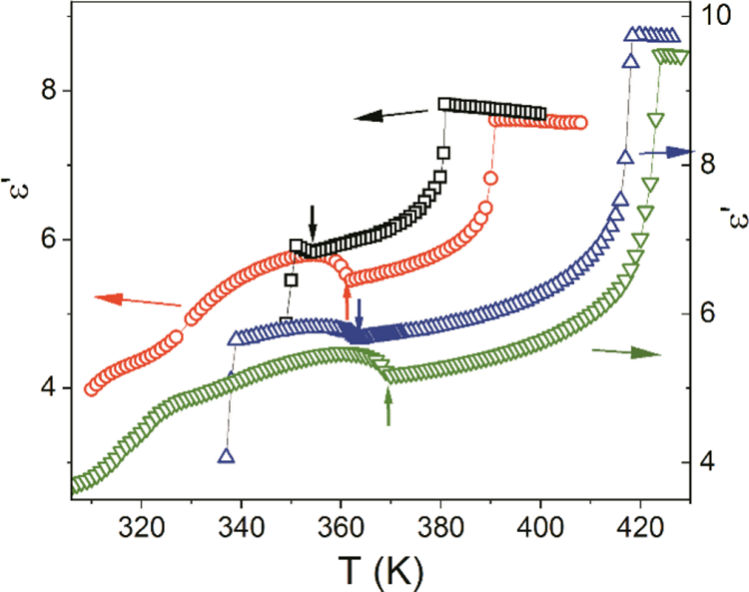
Perpendicular permittivity components: square: CBSC5SCB, circle: CBSC7SCB, inverted triangle: CBSC5OCB, triangle: CBSC7OCB.

The results include the relaxation of the molecular modes and the collective soft mode but do not include the influence of the ionic effects and the collective modes below 10 kHz. In addition, the average dielectric permittivity (ε̅ = (ε_||_ + 2ε_⊥_)/3 and dielectric anisotropy (Δε = ε_||_ – ε_⊥_) are shown in Figure 2 as a function of temperature. The open triangles in Figure 2 represent the static dielectric permittivity that was obtained for the metallic cells that were in homeotropic alignment. The static dielectric permittivity measurements for CBSCnSCB and CBSnOCB exhibited a temperature dependence that was similar to the one that has been reported for other symmetric dimers.^2,6,40^ As was expected for the materials with a positive dielectric anisotropy, the value of the perpendicular component of the permittivity decreased from the isotropic phase after cooling, while the parallel component increased at the isotropic-nematic transition.

**Figure 3:**
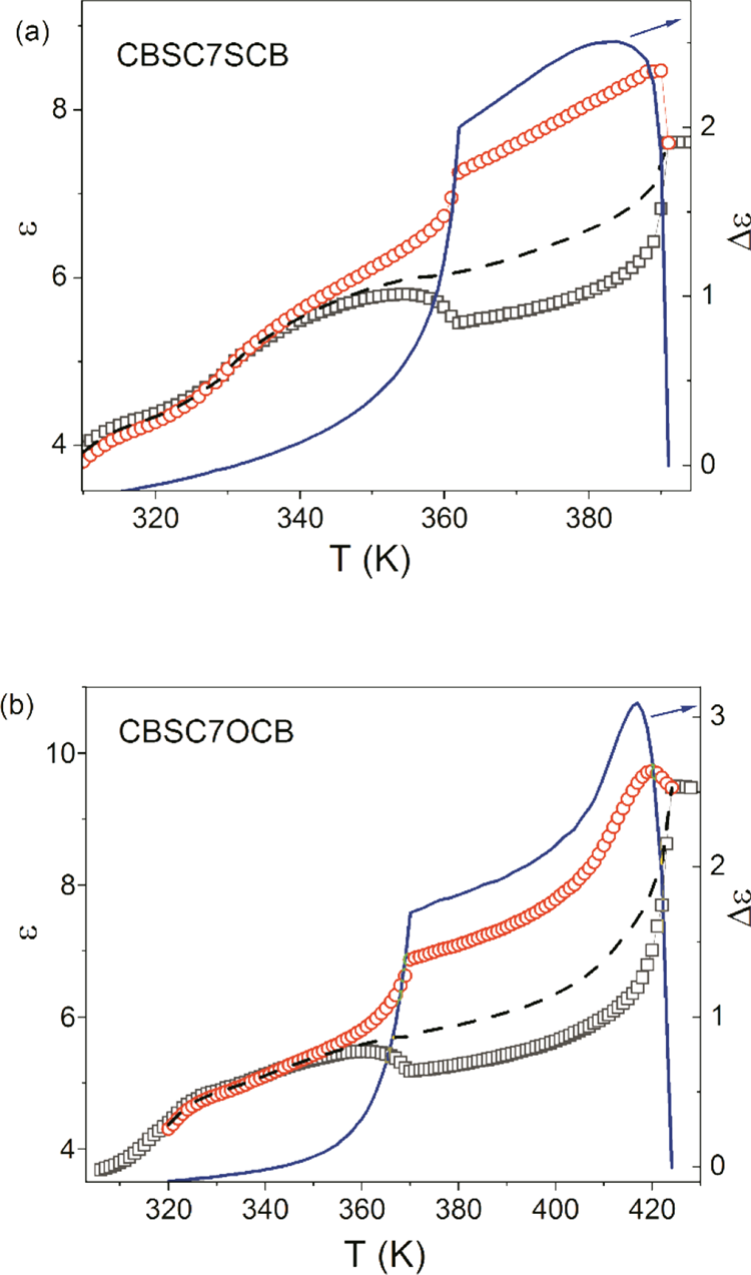
Temperature dependencies of the perpendicular and parallel components of the permittivity (ε_⊥_, ε_||_) that were obtained in planar and homeotropic orientations, respectively, in the presence of a weak measuring field (0.2 Vrms/μm): square: ε_⊥_ perpendicular component, circle: ε_||_ parallel component, dotted line: ε̅ average permittivity (left axis), and line: Δε permittivity anisotropy (right axis). (a) CBSC7CB, (b) CBSC7OCB.

In the case of the odd dimers, this increase can be explained by the contribution of the hairpin conformers at the beginning of the nematic ordering. However, after this initial increase, the parallel permittivity began to decrease in the nematic phase after a further decrease in temperature. As can be observed in Figures 2 and 3, this trend entailed a progressive increase in the difference between the average permittivity and the extrapolated isotropic value as the temperature decreased, that is, a significant decrease in the average dipole moment of the molecule as the order of the phase orientation increased. As was mentioned earlier, this fact can be partially explained by an increase in the population of stretched conformers with the zero longitudinal dipole moment as a result of an increase in the orientation order. After entering the N_TB_ phase, the parallel permittivity component decreased much faster with a decreasing temperature than the perpendicular one, and at a low temperatures, there was a marked reversal of the dielectric anisotropy from positive to negative.

### 4.2 Dynamic of the System: Molecular Modes in the Nematic Phase

The dynamic dielectric responses were measured in metal cells with 50 μm spacer, in the frequency range of 10 Hz to 1 GHz. The dielectric response of CBSCnSCB and CBSCnOCB in the nematic phase exhibited two relaxation processes whose contribution to the dielectric spectrum depended on the orientation of the director in the measurement cell. These dielectric relaxations were related to the rotational diffusion of the dimer molecules: the reorientation of the end-to-end dipolar groups parallel to the director at low frequencies (**m_1_**) according to the theoretical model of dielectric relaxation in nematic dimers^33^ and the precessional motion of the dipolar groups around the director for the high-frequency branch of a spectrum (**m_2_**). In terms of spherical harmonics, their relaxation times are classified to be τ_00_ and τ_10,_ respectively_,_ as defined in eqs 11 and 12.

Due to their symmetry, the bent molecular configurations (trans conformers) for the odd dimers had no longitudinal dipole moment if the angle between the terminal dipoles was greater than 90° and their contribution to the orientational dielectric permittivity was insignificant. However, the hairpin conformers with small interarm angles, for which both rigid units usually tended to align along the director, contributed significantly to the parallel component of the permittivity. Thus, the strong decrease of the permittivity after the temperature was reduced reflected a progressive increase of the population of the bent (trans) molecular conformers, which were accommodated better by the nematic potential.

As was already stated, at higher frequencies, the ε″ spectra were dominated by two maxima. These corresponded to the two molecular relaxation modes, **m_1_** and **m_2_**, of the symmetric CBSCnSCB and asymmetric CBSCnOCB dimers, as was also observed by Cestari et al.,^44^ López et al.,^45^ and Merkel et al.^46^ for CBnCB.

We simultaneously analyzed the relaxation times of the two observed processes in the N phase and were able to describe them unequivocally by the orientational order, *S*, and the so called Debye relaxation time, τ_D,_ which corresponds to the relative relaxation in the absence of orientational order. This can be interpreted simply as an extension of the isotropic relaxation time into the temperature range of the nematic phase. Following eq 4b, the contribution of permittivity to the perpendicular component originated from the precession rotation of the transverse component of the dipole moment, that is, the rotation of the bow axis of the bent-core conformation contributed significantly to the perpendicular component. The higher frequency relaxation process, **m_2_**, can arise from the rotations of a segment of the molecule, that is, the internal rotation of each monomer with the spacer anchored involved the fluctuations of the CB dipolar moment. Because the length of the spacer in between the two mesogens in a dimer is large enough, such an independent internal rotation of each monomer of a dimer is highly feasible. The temperature dependencies of δε_2_ and of the relaxation time of mode **2** are suggestive of the precessional rotation of the longitudinal component of each thioether CB (SCB) dipole moment around the director in a planar-aligned cell.

**Figure 4:**
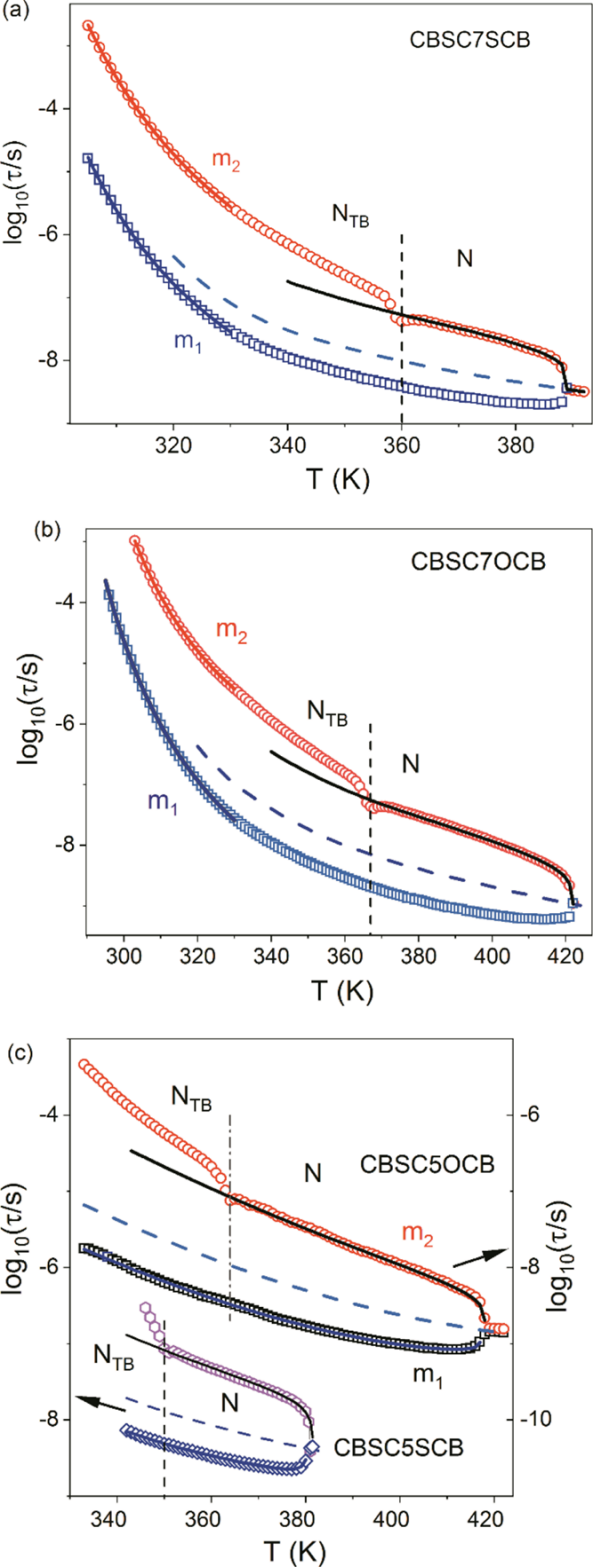
Plots of relaxation times for modes **m_1_** and **m_2_** (5μm planar cell): (a) CBSC7SCB, (b) CBSC7OCB: square, **m_1_** mode; circle, **m_2_** mode, model fitting: dotted line, **m_1_** mode; line, τ_D_ relaxation time, and (c) CBSC5S/OCB: square, **m_1_** mode; circle, **m_2_** mode; diamond, **m_1_** mode; hexagon, **m_2_** mode, model fitting: blue line, **m_1_** mode; black line, **m_2_** mode; dotted line, τ_D_ relaxation time.

The results in the N phase were, however, more complex than for the monomers since the dielectric amplitude and its relaxation rate were strongly affected by the reorganization of the conformers in terms of their molecular shapes. Two major conformers were considered to be the bent cores: a stretched conformer with a transversal dipole moment and a hairpin-shaped conformer with a large longitudinal dipole moment. This was clearly reflected in the results of the temperature dependencies of the average dielectric permittivity (ε̅), which are presented Figures 2 and 3. The strengths δε_1_ and δε_2_ of the **m_1_** and **m_2_** modes (see Figure S1) were strongly affected by both an increase in the population density of the bent conformers as well as the increasing strength of the intermolecular interactions that accompanied the formation of N_TB_ phase. The relaxation times of the modes, however, seemed to be well defined solely by the local orientation order in the nematic phase. For all of the dimers, the dynamic behavior in the nematic phase was reproduced quite well by the molecular dynamic model, as shown in Figure 4a–c.

**Figure 5:**
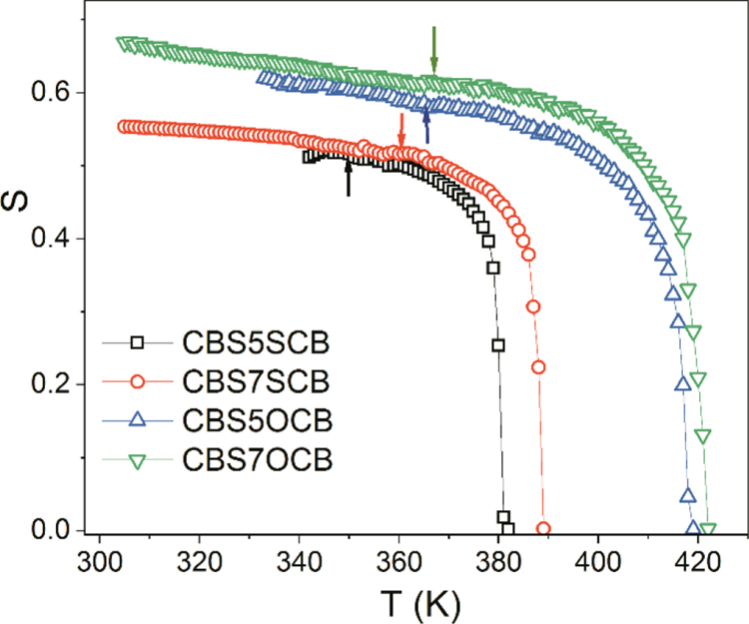
Orientational order parameter, *S*, obtained from fitting the relaxation time of **m_1_** and **m_2_** mode: square: CBSC5SCB, circle: CBSC7SCB, inverted triangle: CBSC5OCB, triangle: CBSC7OCB.

The experimental relaxation times, τ_1_ and τ_2_, for mode **1** and **2**, respectively, were well fitted using eqs 10–12, assuming the uniaxial order parameter, *S*, and τ_D_ were unknown values. The results are shown in Figure 4a–c for τ_D_ and in Figure 5 for the *S*. The temperature dependencies of the orientational parameters quite well correspond to the results obtained by infrared spectroscopy,^15^ but their values are slightly smaller than referenced (IR) because dielectric ones are related to the S-CB or (O-CB) dipole moments, but the IR ones correspond to the molecular (bow) axis.

In the N_TB_ phase, the temperature dependencies of the τ_10_ kept their trends without any step at the transition temperature, despite the fact that the director became tilted with respect to the symmetry axis. It seems that this process is determined exclusively by the local orientational order (relative to the local director), which was growing continuously with a decreasing temperature. If the orientational order is calculated using dielectric strengths or birefringence, then we have *S* in the other reference system: where the *z*-axis is along the helical axis. In such a case, the molecular tilt at the N to N_TB_ transition temperature affects the *S* parameter. The results can be converted from one unit system to another by considering the tilt of the director.

This was not the case for the end-over-end relaxation time (mode **m_1_**). First of all, the relaxation time dependence clearly exhibited a kink at the transition temperature, which indicated an increased relaxation rate at the transition temperature. This was more likely due to critical fluctuations in the director in the vicinity of the transition.^47^ Then, in N_TB_ phase, the temperature trend of the relaxation time suddenly increased with respect to the trend in the nematic phase. We simply interpreted this fact as an apparent growth of the potential barrier, *q*, which separated the two minima along the *n* direction after entering the N_TB_ phase. This finding corresponded well with the orientational correlations effect of the longitudinal dipoles, which was revealed by IR spectroscopy.^16^

**Figure 6:**
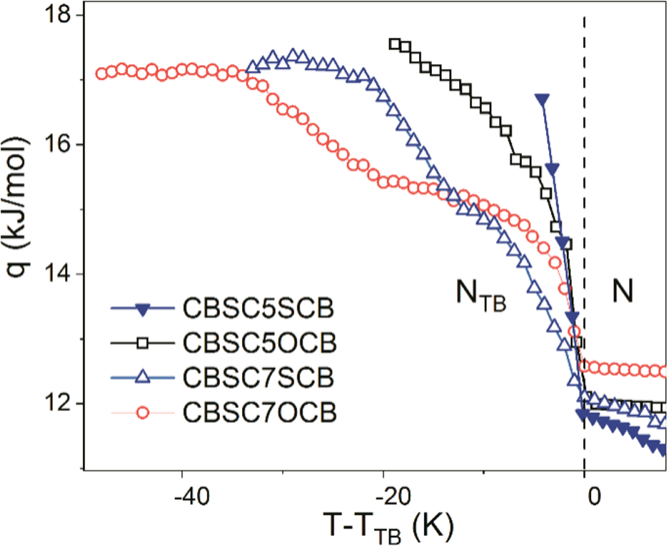
Potential barrier, *q*, separating two minima along *n* direction: solid inverted triangle: CBSC5SCB, square: CBSC5OCB, triangle: CBSC7SOCB, circle: CBSC7OCB.

Figure 6 shows the apparent increase of the potential barrier, *q*, for symmetric and asymmetric dimers on entering the N_TB_ phase. Overall, the *q* barrier grew from about 12 kJ/mol (in N phase) up to about 18 kJ/mol in the N_TB_ phase;, however, the temperature trends for the shorter dimers (*n* = 5) are clearly different than for the longer ones (*n* = 7).

For the shorter linker in dimers, the potential barrier increased steeply, and finally, both samples (CBSC5SCB and CBSC5OCB) soon crystallized at a few degrees below the transition temperature, *T*_TB_, regardless of the cooling rate. Both of the longer linker dimers, CBSC7SCB and CBSC7OCB, exhibited a smaller/slower increase of the potential barriers that extended in the range of 20–30 K, after which they reached their saturation, while the samples underwent vitrification. It is worth mentioning that the barriers increased in two steps: the first step corresponded well to an orientational correlations effect of the longitudinal dipoles (*g*_∥_ < 1) along the major axis of anisotropy, and the second step appeared at the temperature at which the interactions along the perpendicular axis became important (*g*_⊥_ > 1) that was revealed by IR spectroscopy.^16^

We will now focus on the temperature range that is well below (∼20 K) the transition N-N_TB_, during which the typical glass-forming behavior was observed on the cooling for the two longer dimers that had seven carbons in the link (i.e., CBC7SCB and CBSC7OCB). The phenomenological equations, the VFT formula eq 8, were used to describe the temperature dependence of the relaxation time data (τ). The results of the fitting are presented as dashed lines in Figure 4a,b. The VFT parameters that were obtained for eq 8 are listed in Table 1. It should be stressed that two different dielectric glass transition temperatures were obtained as can be observed in Table 1, though they were rather close to one another within about 4 K. It is also interesting to note that the pre-factor τ_0_ for the **m_2_** mode was of the order of 10^–11^ s.

**Table 1:** Fitting Parameters According to Eq 8 for the Different Dimers and the Calculated Glass Transition Temperature for the **m_1_** and **m_2_** Modes

	mode	log_10_[τ(s)]	*T*_0_ (K)	*B* (K)	*T*_gl_ (K)
CBSC7SCB	**m_1_**	–10.1	266.6	682.6	289.8
	**m_2_**	–11.9	265.6	631.9	286.3
CBSC7OCB	**m_1_**	–9.2	261.3	596.0	284.3
	**m_2_**	–11.5	260.5	621.7	280.5

The models that were used to interpret the low frequency relaxation in liquid crystals are often based on a single particle relaxation process; however, spectroscopic probes of molecular motion such as magnetic resonance, neutron scattering, and time-resolved fluorescence depolarization suggest that the reorientation times for mesogens are of the order of 10^–9^ to 10^–10^ s in the isotropic, nematic, and disordered smectic phases. Thus, interpreting the dielectric relaxation processes at the MHz or even kHz frequencies in terms of the rotation of a single molecule is unlikely to be correct. The low frequency relaxations that were observed in liquid crystals are the result of a collective molecular motion, although the models outlined above are useful for analyzing the results and comparing materials.

A more detailed method for studying the dynamics of glass forming behavior is to analyze the derivatives of the relaxation time with respect to the inverted temperature,^48,49^ and the results for CBSC7SCB and CBSC7OCB are presented.^50^

## 5 Conclusions

The dielectric relaxation spectra for two symmetric CBSCnSCB and the asymmetric CBSCnOCB dimers were analyzed in terms of their relaxation processes as predicted by the rotational diffusion model. For the odd dimers when considering a nematic environment, the continuous torsional potential model predicted a broad conformational distribution that was dominated by the bent conformers, but with an appreciable contribution of the hairpin molecular configurations at high temperatures.

The experimental spectra were dominated by the two relaxation processes from the rotations of a segment of a molecule, that is, the internal rotation of each monomer with the spacer anchored that involved the fluctuations of the CB dipolar moment. Because the length of the spacer between the two mesogens in the dimer was large enough, such an independent internal rotation of each monomer of the dimer was highly feasible. By simultaneously analyzing the two processes in the N phase, they were described unequivocally in terms of their orientational order, *S*, and therefore, their isotropic relaxation time, τ_D_, which corresponded the relaxation of the system in the absence of an orientational order. The high frequency relaxation process originated from the precession rotation of the longitudinal component of the dipole moment of the monomers of each SCB dipole moment around the director. The relaxation rate was accelerated with respect to the isotropic relaxation because τ_10_ was shorter compared to τ_D_. The low frequency relaxation originates from the “end-over-end” rotation of the dipole moment of the monomers. The relaxation rate of that mode was lower with respect to the isotropic mode. It was found that the model reproduced both the relaxation times dependence very well in the nematic phase for both modes. The temperature dependencies of the orientational parameters, *S*, that were obtained, corresponded quite well with the results that were obtained with infrared spectroscopy, although their values were slightly smaller because they were related to the S-CB or (O-CB) dipole moments unlike in the case of the IR ones, which described the molecular axis. It is interesting to note that the relaxation time maintained its temperature trend during the transition from the N to N_TB_ phase, as was predicted by the power law approximation of the *S* parameter. It seems that it was determined by the local orientational order, which grew continuously regardless of the tilting of the director that appeared at the transition. The low-frequency relaxation time, τ_00_, was significantly affected by the transition from the N to the N_TB_ phase. Initially, it exhibited a kink at the transition temperature, which was likely due to critical fluctuations of the director in the vicinity of the transition at which the τ_00_ relaxation time began to grow much more steeply. We interpreted this fact as an apparent growth of the potential barrier, *q*, that separated the two minima along the *n* direction after entering the N_TB_ phase. This corresponded well with the effect of the orientational correlations of the longitudinal dipoles, which was revealed using IR spectroscopy.

## Supporting Information Available

Dielectric strength of the relaxation modes **m_1_** and **m_2_** vs temperature for perpendicular alignment (PDF)

## Abbreviations

LC: liquid crystals
N: nematic phase
N_TB_: twist-bend nematic
CB: cyanobiphenyl (CB)
CBCnCB: cyanobiphenyl LC dimers
CBSCnSCB, CBSCnOCB: symmetric and asymmetric thioether/ether dimers
FTIR: Fourier transform infrared spectroscopy
IR: infrared spectroscopy
AFM: Atomic Force Microscopy
NMR: Nuclear Magnetic Resonance
FFTEM: Freeze Fracture Transmission Electron Microscope
m_1_: low-frequency mode
m_2_: high-frequency mode
*T*_g_: glass transition
Δε: anisotropy of dielectric permittivity
μ: the transition dipole
*S* and *D*: uniaxial and biaxial order parameters
*E*_a_: activation energy
AG: Adam-Gibbs entropy model
VFT: Vogel–Fulcher–Tammann model
