# Supplementary material for: Dielectric Study of Liquid Crystal Dimers: Probing the Orientational Order and Molecular Interactions in Nematic and Twist-Bend Nematic Phases
Source: J Phys Chem B. 2023 Aug 1;127(31):7082–90. doi: 10.1021/acs.jpcb.3c03496 (PMC10424235; doi:10.1021/acs.jpcb.3c03496)
Supplement: Supplementary file 1 — jp3c03496_si_001.pdf [file jp3c03496_si_001.pdf]

Supporting Information

Dielectric Study of Liquid Crystal Dimers: Probing the Orientational Order and Molecular Interactions in Nematic and Twist-Bend Phases

Antoni Kocot[1‡], Małgorzata Czarnecka[2], Yuki Arakawa[3] and Katarzyna Merkel[1‡]*

AUTHOR ADDRESS: [1]Institute of Materials Engineering, Faculty of Science and Technology, University of Silesia, 75 Pułku Piechoty 1a, Chorzów 41-500, Poland

[2]Faculty of Electrical Engineering, Automatics, Computer Science and Biomedical Engineering, AGH University of Science and Technology, al. Adama Mickiewicza 30, Cracow, 30-059 Poland

[3]Department of Applied Chemistry and Life Science, Graduate School of Engineering, Toyohashi University of Technology, Toyohashi 441-8580, Japan

KEYWORDS: Liquid crystal dimers, Polar Nematics, Ferroelectric Nematics, Dielectric Spectroscopy, Molecular and Collective Modes, Dynamics

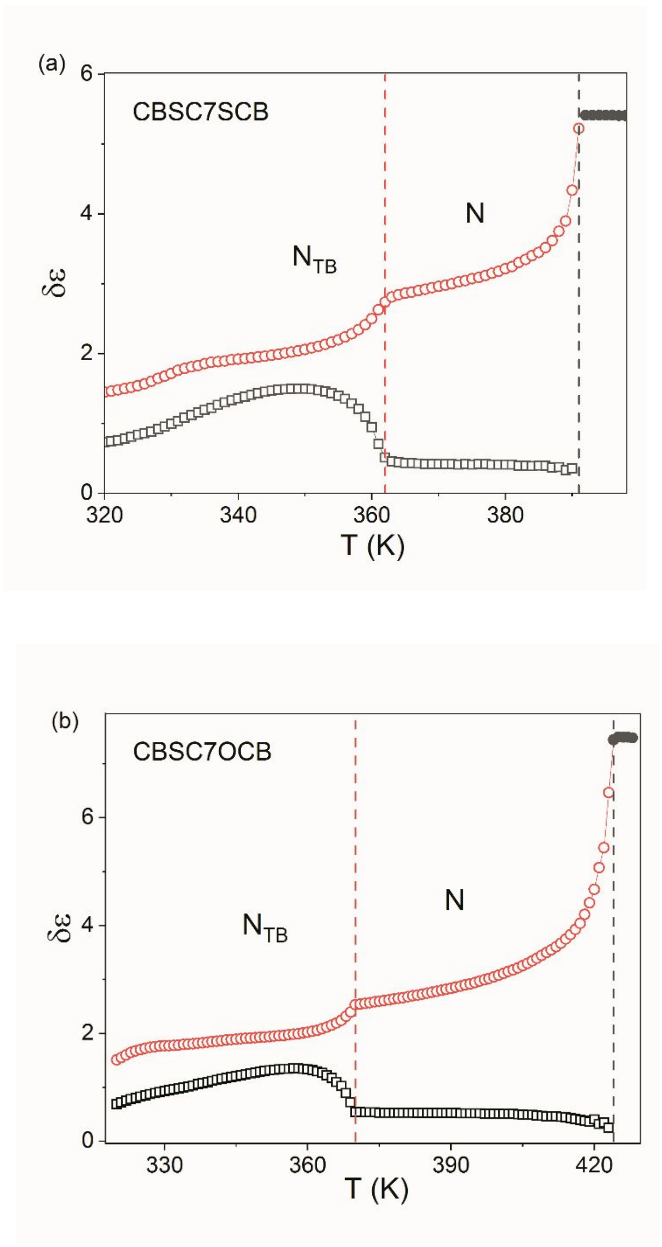

Figure S1. Dielectric strength of the relaxation modes $\mathbf{m_1}$ and $\mathbf{m_2}$ vs temperature for perpendicular alignment; (📪) isotropic phase, (✂) low-frequency mode $\mathbf{m_1}$ in the N and N_{TB} phases, (📫) high-frequency mode, $\mathbf{m_2}$, in the N and N_{TB} phases. (a) CBSC7SCB, (b) CBSC7OCB.
